# Differential microRNA expression following infection with a mouse-adapted, highly virulent avian H5N2 virus

**DOI:** 10.1186/s12866-014-0252-0

**Published:** 2014-09-30

**Authors:** Eun-Ji Choi, Hyeun Bum Kim, Yun Hee Baek, Eun-Ha Kim, Philippe Noriel Q Pascua, Su-Jin Park, Hyeok-il Kwon, Gyo-Jin Lim, Semi Kim, Young-Il Kim, Young-Ki Choi

**Affiliations:** College of Medicine and Medical Research Institute, Chungbuk National University, 12 Gaeshin-Dong Heungduk-Ku, Cheongju, 361-763 Republic of Korea; Department of Animal Resources Science, Dankook University, Dandae-ro 119, Cheonan, 330-714 Republic of Korea

**Keywords:** Influenza A virus, MicroRNA, Inhibitor, Virulence, Replication

## Abstract

**Background:**

MicroRNAs (miRNAs) are known to regulate various biological processes, including expression of cellular gene and virus-induced inflammation. Recently, studies have indicated that some miRNAs could regulate influenza virus replication. Due to differential sensitivities of influenza A virus strains to different species (avian and mammalian), variations in host responses may be observed. Therefore, we investigated and compared the differences in global host miRNA expression in mouse lungs infected with wild type low pathogenicity A/Aquatic bird/Korea/w81/2005 (H5N2) (w81) or mouse-adapted virulent A/Aquatic bird /Korea/ma81/2007 (H5N2) (ma81) virus.

**Results:**

Although the mice infected with ma81 exhibited much greater mortality than w81-infected mice, the parental w81 virus induced a higher number of differentially expressed miRNAs compared to the ma81 virus. Between these 2 viruses, a total of 27 and 20 miRNAs were commonly expressed at 1 dpi and 3 dpi, respectively. It is noteworthy that only 9 miRNAs (miR-100-5p, miR-130a-5p, miR-146b-3p, miR-147-3p, miR-151-5p, miR-155-3p, miR-223-3p, miR-301a-3p, and miR-495-3p) were significantly upregulated in both lungs infected with either wild type w81 or the mouse-adapted ma81 strain at both time points. Notably, expression levels of miR-147-3p, miR-151-5p, miR-155-3p, and miR-223-3p were higher in the lungs of mice infected with the ma81 virus than those infected with the w81 virus. To identify potential roles of these miRNAs in regulating influenza virus replication, each group of mice was intranasally treated with each inhibitor of specifically targeting 4 miRNAs, and then challenged with 5 mouse lethal dose 50% (MLD_50_) of the virulent ma81 virus on the following day. Although the specific miRNA inhibitors could not completely attenuate mortality or reduce viral replication, the miR-151-5p- and miR-223-3p-inhibitors reduced mortality of inoculated mice to 70% and substantially delayed death.

**Conclusions:**

Our results suggest that the mammalian adaptation of avian influenza A virus results in a different miRNA expression pattern in lungs of virus-infected mice compared with its parental strain, and use of specific miRNA inhibitors to target genes associated with the immune response or cell death may affect virulence and virus replication.

**Electronic supplementary material:**

The online version of this article (doi:10.1186/s12866-014-0252-0) contains supplementary material, which is available to authorized users.

## Background

Influenza A viruses belong to the Orthomyxoviridae family of RNA viruses and are a persistent cause of respiratory diseases in animals and humans [1]. Variants of these viruses have been isolated from a broad range of hosts, including chickens, pigs, human, horses, domestic ducks, and migrant shorebirds [2,3]. They have a single negative-sense RNA genome packed into 8 gene segments that encode 11 or 12 viral proteins; however, recent studies suggest that more viral proteins may be produced by some strains [4-6]. Constant evolution of influenza viruses may occur through a number of mechanisms, including antigenic drift, genetic shift, defective-interfering particles, and RNA molecular recombination [7], and global virus pandemics caused by transmission of novel viruses, such as the 1918 influenza pandemic, have led to disastrous outcomes [8]. Activation of the host innate immune system in response to influenza infection triggers phagocytosis for viral pathogen elimination; however, the antiviral response to infection is sometimes ineffective or even detrimental in the host. In fact, studies have implicated the host innate immune system as the cause of severe influenza virulence [9,10], including the abnormal innate immune response responsible for the atypical virulence of the 1918 pandemic influenza virus [11] and the highly pathogenic H5N1 variant [9,12,13].

MicroRNAs (miRNAs) are noncoding RNAs 20–22 nucleotides long that bind target miRNAs to cause their degradation or translational inhibition and thereby regulate various biological processes [14]. Recently, studies have implicated miRNAs in viral replication and have indicated they can both inhibit and promote viral infections [15,16]. Expression of miRNAs has been reported in response to several viruses, such as human immunodeficiency virus-1, simian immunodeficiency virus [17,18], hepatitis B virus [19], hepatitis C virus [20], Epstein-Barr virus [21], and oncogenic human papillomaviruses [22]. Furthermore, miRNA expression patterns have been profiled in mouse lung and A549 cells infected with pandemic influenza virus [23,24]. Additionally, differential expression of miRNAs has been observed in various animals, including H5N1 influenza virus-infected cynomolgus macaque lungs [25] and mouse lungs [26], H1N2 virus-infected pigs [27], and avian H5N3 influenza virus-infected chickens [28,29]. Such studies provide evidence that miRNAs play an important role during influenza virus infection. Moreover, recent studies have indicated that some cellular miRNAs can inhibit influenza virus replication or propagation [30,31].

Studies have shown that the acquisition of virulence in new host through mouse adaptation is associated with mutations in various gene segments [32-37]. Commonly identified virulence markers include E627K in PB2 and the multibasic cleavage site motif in HA, in addition to mutated PB1-F2 and NS1 proteins [38]. The polymerase gene, PB2 gene, is an important determinant of virulence in the HPAI H5N1 and H7N7 viruses [39,40].

In our previous study to investigate the molecular changes that occur during adaptation of a low pathogenic avian influenza virus subtype to a mammalian host, we serially passaged a wild bird H5N2 isolate, A/Aquatic bird/Korea/w81/05 (w81), in the lungs of mice. In contrast to the parent strain, the resulting mouse-adapted strain (ma81) was both highly pathogenic and lethal [41]. Full length sequencing results showed that nonconserved mutations were observed in six viral genes (those for PB2, PB1, PA, HA, NA, and M) of w81 resulting in ma81. However, reverse genetic experiments substituting viral genes and mutations demonstrated that the PA gene was a determinant of the enhanced virulence in mice, and that a Thr-to-Iso substitution at position 97 of PA played a key role [41]. In growth kinetics studies, ma81 showed enhanced replication in mammalian cell lines, and a PA97I mutation in w81 was identified to cause such replication. Because influenza A virus strains have different sensitivities to different mammalian hosts (i.e., avian versus mammalian strains), it is possible that different host responses may be observed during infection with wild type or mammalian-adapted-avian influenza (AI) virus strains even though they share the same genetic backbones. Therefore, in the present study, we compared miRNA expression profiles in the lungs of mice infected with wild type, low virulence, avian parental w81 (H5N2) virus or the mouse-adapted highly virulent ma81 strain to investigate whether mammalian adaptation of the avian influenza virus could differentially alter the expression of cellular miRNAs. Specifically, miRNAs were assessed at 1 and 3 days post infection (dpi), and 27 and 20 miRNAs were differentially expressed by both viruses at 1 and 3 dpi, respectively, even though many miRNAs were found to be commonly induced by both viruses. These results suggested that mammalian adaptation of avian influenza A virus can alter the host miRNA expression pattern in lungs of virus-infected mice, and thus these molecules might play important roles in viral replication and pathogenesis. In addition, our results present the feasibility of using miRNAs as therapeutic targets in the face of a mammalian-adapted, avian influenza virus infection as inhibition of certain miRNAs reduced viral replication and increased survival in the mouse model.

## Results

### Virus titration and clinical manifestations of wild type w81 and mouse-adapted variant ma81

**Figure 1:**
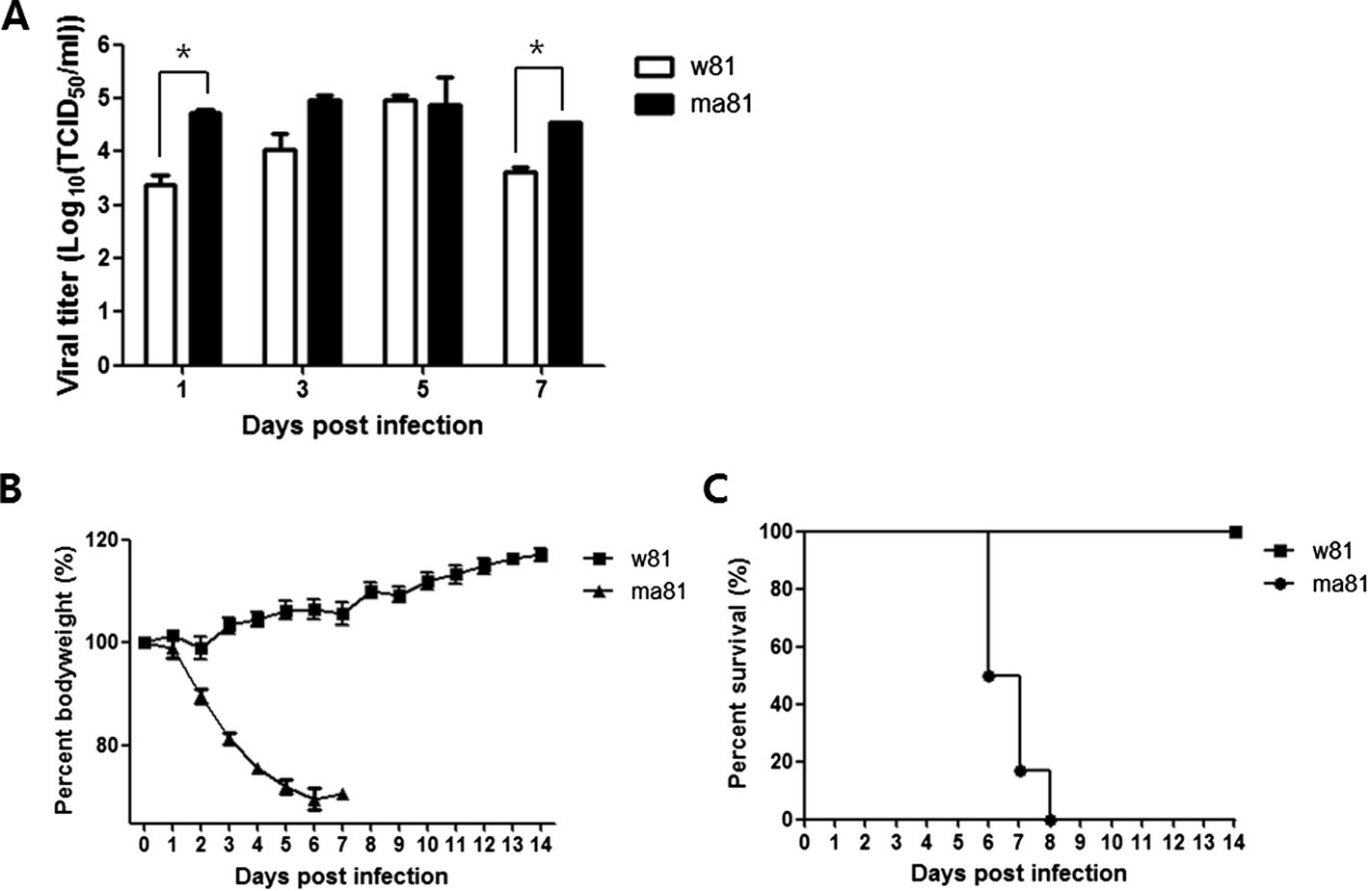
**Comparison of virulence between w81 and ma81 virus infection in C57BL/6 mice.** Groups of 18 C57BL/6 mice were inoculated intranasally with 5 MLD50 of wild type (w81) or mouse-adapted (ma81) H5N2 avian influenza virus. Viral growth kinetics in mouse lungs were determined by TCID50 at 1, 3, 5, and 7 dpi **(A)**. Data are expressed as mean ± SD titers from 3 lungs per time point. Mean body weight changes **(B)** and survival rates **(C)** were monitored in six remaining mice for 14 days. *, P value ≤0.05; MLD50, mouse lethal dose 50%.

To compare the pathogenicity of each virus in infected mice, clinical symptoms, and survival rates were measured for 14 dpi and lung viral titers were evaluated at 1, 3, 5, and 7 dpi. The lungs harvested from mice infected with a mouse-adapted variant ma81 showed relatively higher virus titers ranging from 10^4.05^ to 10^5.8^ 50% tissue culture infectious doses (TCID_50_/ml) compared to lung tissues obtained from those inoculated with a wild type w81 virus which produced titers ranging from 10^3.05^ to 10^5.05^ TCID_50_/ml (Figure 1A). Overall, the ma81 strain replicated more efficiently in infected mouse lungs than the wild type w81 strain.

All mice infected with ma81 showed rapid body weight losses and significant disease symptoms, such as hunched back, ruffled fur, and lethargy until succumbing to death within 8 dpi. In contrast, no clinical signs of influenza, including weight loss and death, were evident in mice infected with the wild type w81 during the experimental period, and all mice inoculated with w81 survived until the end of the experiment (Figure 1B and C). These results clearly demonstrate that mouse-adapted ma81 is more virulent and more lethal than the parental w81 strain.

### Differential miRNA expression in mouse lungs infected with either wild type w81 or mouse-adapted variant ma81

To investigate the changes in the host gene expression profile occurring during adaptation of an avian influenza virus subtype to a mammalian host, global cellular miRNA expression patterns in mouse lungs infected with either w81 or ma81 were compared with those of uninfected controls. In this study, only differentially expressed miRNAs with a P < 0.05 and fold change > 2 are described.

**Figure 2:**
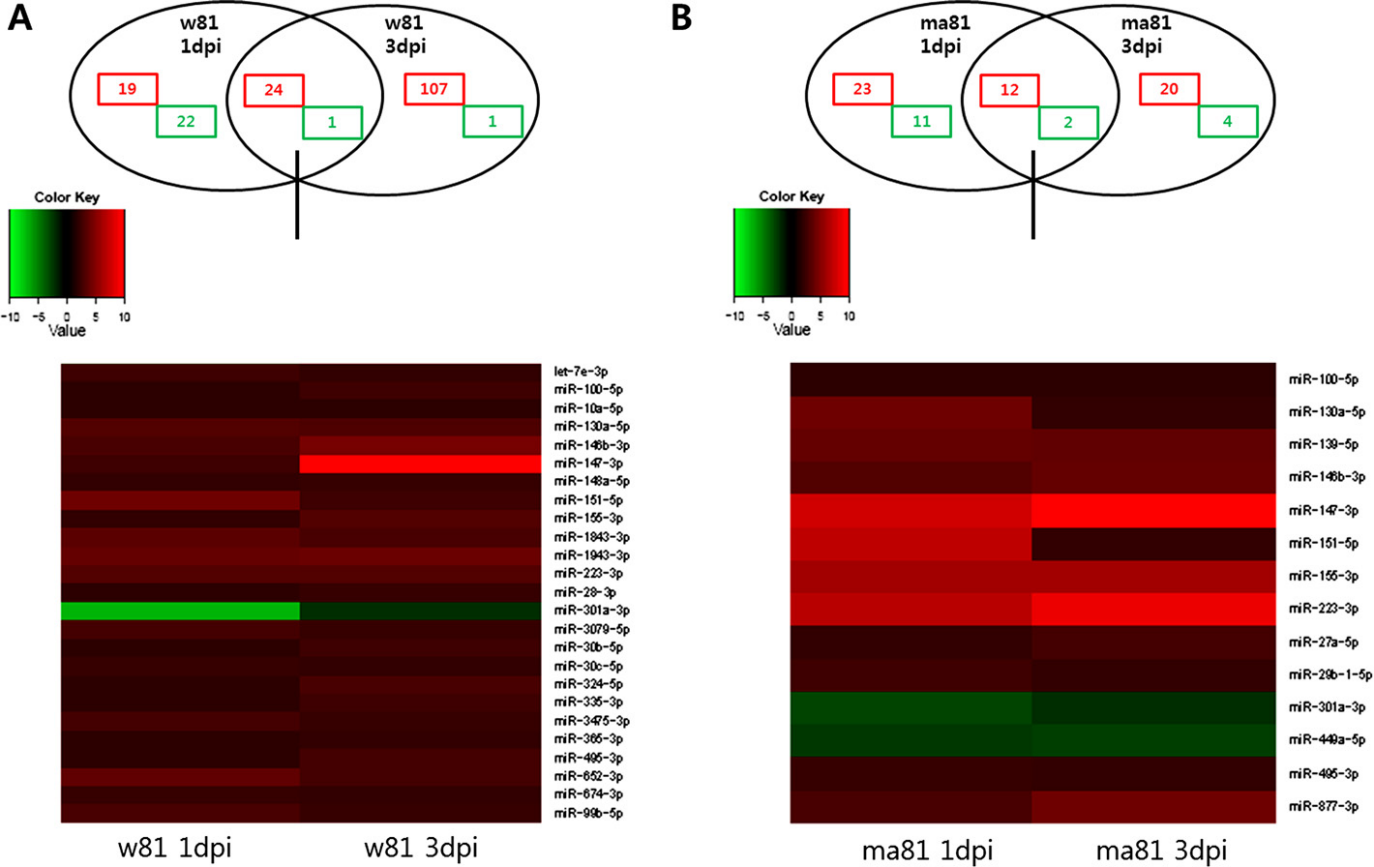
**Differentially expressed miRNAs in virus-infected mouse lungs at 1 dpi and 3 dpi.** Venn diagram and heat maps indicate expressed miRNAs during w81 or ma81 infections relative to the mock infection (control). Red and green colors represent up- and down-regulation, respectively. Fold change ≥2 or ≤ −2, P value ≤0.05. **A**: w81-infected mouse lungs, **B**: ma81-infected mouse lungs.

A total number of differentially expressed miRNAs in mouse lungs infected with w81 at 1 dpi and 3 dpi were presented in Figure 2A. In the lungs of mice infected with wild type w81 at 1 dpi, 43 miRNAs were differentially upregulated, and 23 miRNAs were downregulated (the circle to the left of the venn diagram in Figure 2A). At 3 dpi, a total of 131 miRNAs were differentially upregulated, and 2 miRNAs were downregulated (the circle to the right of the venn diagram in Figure 2A). Altogether, the expression of 174 miRNAs was significantly altered at both 1 and 3 dpi. 24 upregulated and 1 downregulated miRNAs were commonly found at both 1 and 3 dpi (the overlapping portion of the venn diagram in Figure 2A). When infected with mouse-adapted ma81, a relatively small number of differentially expressed miRNAs were identified in the lung (Figure 2B) with a total of only 72 miRNAs being differentially expressed. At 1 dpi, 35 miRNAs were upregulated while 13 miRNAs were downregulated (the circle to the left of the venn diagram in Figure 2B). At 3 dpi, 32 miRNAs were upregulated, and 6 miRNAs were downregulated (the circle to the right of the venn diagram in Figure 2B). Of the 72 differentially expressed miRNAs, only 12 miRNAs were upregulated and 2 miRNAs were downregulated at both 1 and 3 dpi (the overlapping portion of the venn diagram in Figure 2B).

Differentially expressed miRNAs identified to be commonly presented in virus-infected mouse lungs at both 1 dpi and 3 dpi were presented in Tables 1 (mouse lungs infected with w81) and 2 (mouse lungs infected with ma81). It is important to note that only 9 miRNAs (miR-100-5p, miR-130a-5p, miR-146b-3p, miR-147-3p, miR-151-5p, miR-155-3p, miR-223-3p, miR-301a-3p, and miR-495-3p) were significantly upregulated or downregulated in both lungs infected with either wild type w81 or the mouse-adapted ma81 strain at all time points (Tables 1 and 2). Infection with ma81 but not w81 resulted in the unique upregulation of miR-139-5p, miR-27a-5p, miR-29b-1-5p, and miR-877-3p and the downregulation of miR-449a-5p at both 1 and 3 dpi (Tables 1 and 2).

**Table 1:** **Differentially expressed miRNAs identified to be commonly presented in w81-infected mouse lungs at both 1 dpi and 3 dpi**

**miRNA**	**w81‡**			
	**1dpi**		**3dpi**	
	Fold change*	**P value§**	**Fold change**	**P value**
let-7e-3p	2.6	0.008	2.3	0.006
miR-100-5p†	2.1	0.005	2.6	0
miR-10a-5p	2.1	0.009	2	0.002
miR-130a-5p†	3.4	0.036	3.2	0.01
miR-146b-3p†	3.1	0.014	4.7	0.003
miR-147-3p†	2.7	0.013	22.8	0.012
miR-148a-5p	2.3	0.029	2.4	0.002
miR-151-5p†	4.5	0.013	2.7	0.014
miR-155-3p†	2.3	0.015	3.5	0.016
miR-1843-3p	3.6	0.022	3.1	0.007
miR-1943-3p	4	0.01	4.3	0.049
miR-223-3p†	3.5	0.037	3.4	0.009
miR-28-3p	2	0.021	2.4	0.001
miR-301a-3p†	−6.9	0.019	−2	0.048
miR-3079-5p	2.8	0.035	2.4	0.034
miR-30b-5p	2	0.024	2.6	0.007
miR-30c-5p	2.5	0.038	2.2	0.024
miR-324-5p	2.1	0.02	3.1	0
miR-335-3p	2.1	0.004	2.7	0.003
miR-3475-3p	2.8	0.019	2.5	0.008
miR-365-3p	2.1	0.012	2.2	0.016
miR-495-3p†	2	0.042	2.9	0.01
miR-652-3p	3.9	0.042	2.8	0.011
miR-674-3p	2.5	0.019	2.2	0.001
miR-99b-5p	3.1	0.007	2.5	0

‡Global cellular miRNA expression patterns in mouse lungs infected with w81 were compared with those of uninfected control mouse lungs. Differentially expressed miRNAs with a P < 0.05 and fold change > 2 are described.

*Sequence reads were normalized to determine the number of transcripts per million (TPM) using the following formula: Normalized expression = Actual miRNA count/Total count of clean reads*1000000. Then, fold changes of miRNAs were evaluated using the following formula: Fold change = log2 (treatment/control).

§P-value was calculated from the normalized expression values as previously described [66].

†Significantly upregulated in both lungs infected with either wild type w81 or the mouse-adapted ma81 strain at all time points.

**Table 2:** **Differentially expressed miRNAs identified to be commonly presented in ma81-infected mouse lungs at both 1 dpi and 3 dpi**

**miRNA**	**ma81‡**			
	**1dpi**		**3dpi**	
	Fold change*	**P value§**	**Fold change**	**P value**
miR-100-5p†	2	0.035	2	0.007
miR-130a-5p†	4.5	0.001	2.2	0.03
miR-139-5p	4	0.031	3.8	0.003
miR-146b-3p†	3.4	0.011	4.1	0.046
miR-147-3p†	7.8	0.045	25.2	0.04
miR-151-5p†	7.3	0.027	2.2	0.002
miR-155-3p†	6.2	0.024	6.2	0.018
miR-223-3p†	7.1	0.005	8.9	0.013
miR-27a-5p	2.2	0.035	2.9	0.023
miR-29b-1-5p	2.7	0.043	2.2	0.021
miR-301a-3p†	−2.8	0.042	−2	0.049
miR-449a-5p	−2.5	0.046	−2.7	0.016
miR-495-3p†	2.4	0.039	2.3	0.004
miR-877-3p	3.1	0.04	4.4	0.04

‡Global cellular miRNA expression patterns in mouse lungs infected with ma81 were compared with those of uninfected control mouse lungs. Differentially expressed miRNAs with a P < 0.05 and fold change > 2 are described.

*Sequence reads were normalized to determine the number of transcripts per million (TPM) using the following formula: Normalized expression = Actual miRNA count/Total count of clean reads*1000000. Then, fold changes of miRNAs were evaluated using the following formula: Fold change = log2 (treatment/control).

§P-value was calculated from the normalized expression values as previously described [66].

†Significantly upregulated in both lungs infected with either wild type w81 or the mouse-adapted ma81 strain at all time points.

### Distinctions in miRNA expression between wild type w81 and mouse-adapted variant ma81 infections

**Figure 3:**
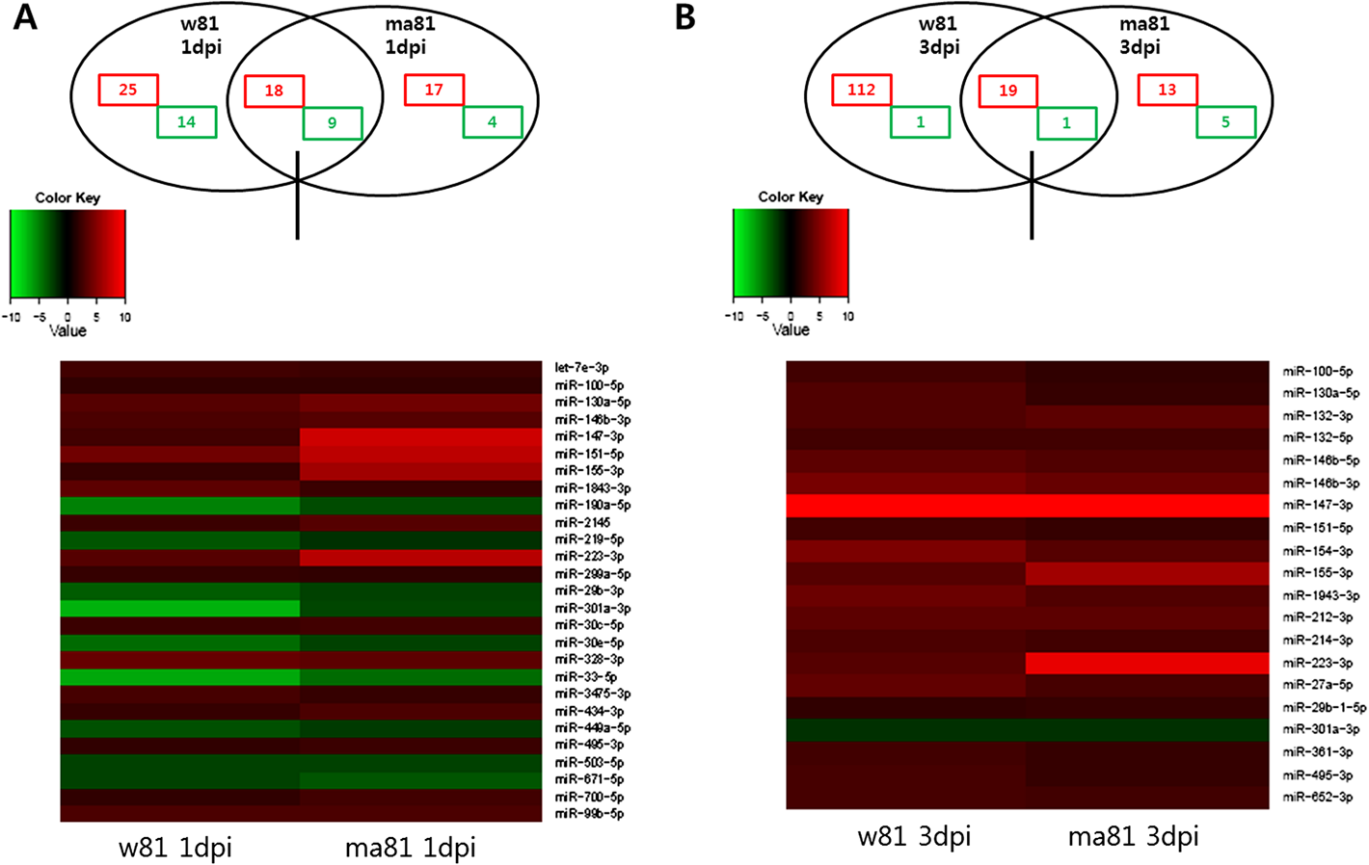
**Comparison of differentially expressed miRNAs in w81-infected and ma81-infected mouse lungs at 1 dpi and 3 dpi.** Venn diagram and heat maps indicate expressed miRNAs during w81 and ma81 infections relative to the mock infection (control) on 1 and 3 dpi. Red and green colors represent up- and down-regulation, respectively. Fold change ≥2 or ≤ −2, P value ≤0.05. **A**: 1dpi, **B**: 3dpi.

Differentially expressed miRNAs between mouse lungs infected either with w81 or ma81 were compared (Figure 3). When comparing the miRNA expression profiles in lungs infected with either wild type w81 or mouse-adapted variant ma81, it became apparent that the number of differentially expressed miRNAs increased over time compared to the controls. Specifically, there were 87 (Figure 3A) and 151 (Figure 3B) differentially expressed miRNAs detected at 1 and 3 dpi, respectively. Out of 87 differentially expressed miRNAs, only 18 upregulated and 9 downregulated miRNAs were commonly found at 1 dpi with both viruses (the overlapping portion of the venn diagram in Figure 3A). Similarly, at 3 dpi, 19 out of 151 differentially expressed miRNAs were commonly upregulated, and only 1 miRNA was commonly downregulated (the overlapping portion of the venn diagram in Figure 3B). Twenty-seven and 20 differentially expressed miRNAs identified to be commonly presented at 1 and 3 dpi were presented in Tables 3 and 4. Of these, only miR-100-5p, miR-130a-5p, miR-146b-3p, miR-147-3p, miR-151-5p, miR-155-3p, miR-223-3p, miR-301a-3p, and miR-495-3p were commonly upregulated at both 1 and 3 dpi.

**Table 3:** **Differentially expressed miRNAs identified to be commonly presented in mouse lungs infected either with w81 or ma81 at 1 dpi**

**miRNA**	**w81‡**		**ma81‡**	
	**1dpi**		**1dpi**	
	Fold change*	**P value§**	**Fold change**	**P value**
let-7e-3p	2.6	0.008	2.5	0.048
miR-100-5p†	2.1	0.005	2	0.035
miR-130a-5p†	3.4	0.036	4.5	0.001
miR-146b-3p†	3.1	0.014	3.4	0.011
miR-147-3p†	2.7	0.013	7.8	0.045
miR-151-5p†	4.5	0.013	7.3	0.027
miR-155-3p†	2.3	0.015	6.2	0.024
miR-1843-3p	3.6	0.022	2.5	0.046
miR-190a-5p	−5	0.033	−3.1	0.045
miR-2145	2.5	0.017	3.5	0.024
miR-219-5p	−3.5	0.005	−2	0.039
miR-223-3p†	3.5	0.037	7.1	0.005
miR-299a-5p	2.3	0.004	2.1	0.018
miR-29b-3p	−3.6	0.012	−2.7	0.047
miR-301a-3p†	−6.9	0.019	−2.8	0.042
miR-30c-5p	2.5	0.038	2.6	0.035
miR-30e-5p	−4.3	0	−2.7	0.022
miR-328-3p	4.1	0.011	3.7	0.034
miR-33-5p	−6.5	0.024	−4.2	0.042
miR-3475-3p	2.8	0.019	2.3	0.011
miR-434-3p	2.3	0	3	0
miR-449a-5p	−3.2	0.016	−2.5	0.046
miR-495-3p†	2	0.042	2.4	0.039
miR-503-5p	−2.6	0.007	−2.6	0.029
miR-671-5p	−2.6	0.038	−3.5	0.017
miR-700-5p	2	0.003	2.6	0.026
miR-99b-5p	3.1	0.007	3.1	0.032

‡Global cellular miRNA expression patterns in mouse lungs infected either with w81 or ma81 were compared with those of uninfected control mouse lungs. Differentially expressed miRNAs with a P < 0.05 and fold change > 2 are described.

*Sequence reads were normalized to determine the number of transcripts per million (TPM) using the following formula: Normalized expression = Actual miRNA count/Total count of clean reads*1000000. Then, fold changes of miRNAs were evaluated using the following formula: Fold change = log2 (treatment/control).

§P-value was calculated from the normalized expression values as previously described [66].

†Commonly upregulated at both 1 and 3 dpi.

**Table 4:** **Differentially expressed miRNAs identified to be commonly presented in mouse lungs infected either with w81 or ma81 at 3 dpi**

**miRNA**	**w81‡**		**ma81‡**	
	**3dpi**		**3dpi**	
	Fold change*	**P value§**	**Fold change**	**P value**
miR-100-5p†	2.6	0	2	0.007
miR-130a-5p†	3.2	0.01	2.2	0.03
miR-132-3p	3.3	0.01	3.6	0
miR-132-5p	2.6	0.015	2.7	0.049
miR-146b-5p	3.6	0.004	3.2	0.009
miR-146b-3p†	4.7	0.003	4.1	0.046
miR-147-3p†	22.8	0.012	25.2	0.04
miR-151-5p†	2.7	0.014	2.2	0.002
miR-154-3p	4.8	0.001	3.5	0.031
miR-155-3p†	3.5	0.016	6.2	0.018
miR-1943-3p	4.3	0.049	3.3	0.033
miR-212-3p	3.7	0	3.6	0.048
miR-214-3p	3	0.007	2.6	0.026
miR-223-3p†	3.4	0.009	8.9	0.013
miR-27a-5p	3.9	0.001	2.9	0.023
miR-29b-1-5p	2.1	0.037	2.2	0.021
miR-301a-3p†	−2	0.048	−2	0.049
miR-361-3p	2.6	0.032	2.3	0.009
miR-495-3p†	2.9	0.01	2.3	0.004
miR-652-3p	2.8	0.011	2.7	0.032

‡Global cellular miRNA expression patterns in mouse lungs infected either with w81 or ma81 were compared with those of uninfected control mouse lungs. Differentially expressed miRNAs with a P < 0.05 and fold change > 2 are described.

*Sequence reads were normalized to determine the number of transcripts per million (TPM) using the following formula: Normalized expression = Actual miRNA count/Total count of clean reads*1000000. Then, fold changes of miRNAs were evaluated using the following formula: Fold change = log2 (treatment/control).

§P-value was calculated from the normalized expression values as previously described [66].

†Commonly upregulated at both 1 and 3 dpi.

Among the 238 differentially expressed miRNAs, we selected 4 miRNAs with greater than 2-fold differences in expression levels in the lungs of mice that were infected with either w81 or ma81 compared to mock control infections for in-depth analyses (miRNAs are miR-151-5p, miR-223-3p, miR-147-3p, and miR-155-3p) (Table 5). These 4 differentially expressed miRNAs identified to be commonly presented in mouse lungs infected either with w81 or ma81 at both 1 and 3 dpi. Notably, expression levels of miR-151-5p, miR-223-3p, miR-147-3p, and miR-155-3p were higher in the lungs of mice infected with the ma81 virus than those infected with the w81 virus. These 4 miRNAs were tested to investigate the potential roles of miRNAs in virus replication. To verify the expression patterns of the 4 selected differentially expressed miRNAs in lungs, real-time RT-PCR was conducted, and the similar results (w81, ma81) confirmed the upregulation of all 4 miRNAs with deep sequencing analysis in terms of direction of regulation at each time point (Additional file 1).

**Table 5:** **Potential functions of the investigated miRNAs**

**ID**	**w81**		**ma81**		**functions**
	**1dpi**	**3dpi**	**1dpi**	**3dpi**	
miR-147-3p	2.7	22.8	7.8	25.2	Inducing toll-like receptor stimulation and regulating murine macrophage inflammatory responses [49]
miR-151-5p	4.5	2.7	7.3	2.2	Regulating in tumor cell migration and spreading of hepatocellular carcinoma [50]
miR-155-3p	2.3	3.5	6.2	6.2	May inhibit malignant growth, viral infections [51,52,54,55]
miR-223-3p	3.5	3.4	7.1	8.9	Promoting granulocytic differentiation [53,56]

### Gene ontology analysis

**Figure 4:**
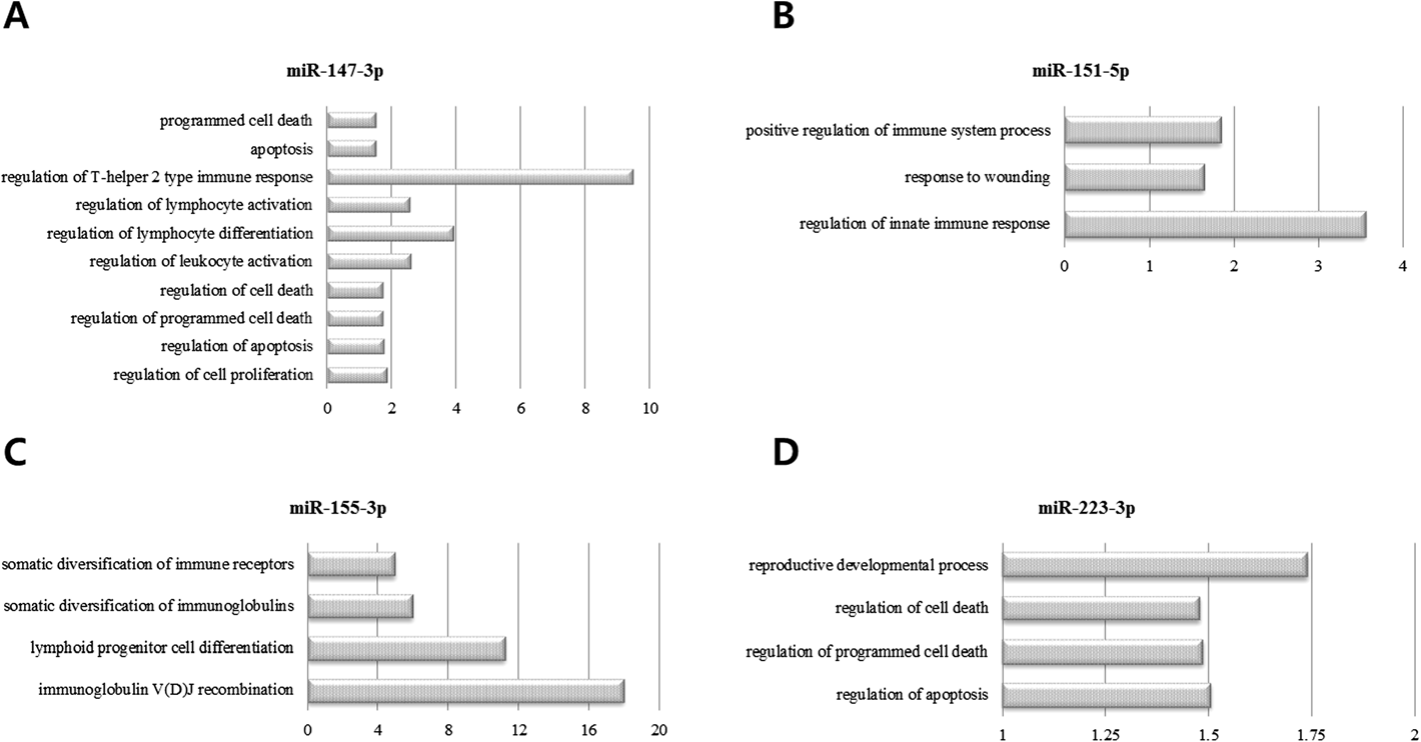
**GO terms of molecular function involved in cell death or immunity or inflammatory responses for the predicted targets of the selected differentially expressed miRNAs.** Potential targets of each of 4 selected miRNAs were predicted using miRanda version 3.0. These predicted targets were subjected to GO analysis using DAVID version 6.7. **A**: miR-147-3p, **B**: miR-151-5p, **C**: miR-155-3p, **D**: miR-233-3p. X-axis represents fold enrichment.

In order to predict the roles of the selected differentially expressed miRNAs in response to w81 and ma81 influenza virus infection, potential targets of each of 4 selected miRNAs were predicted using miRanda version 3.0. A total of 3763 predicted targets were obtained for the differentially expressed miRNAs. These predicted targets were subjected to GO analysis using DAVID version 6.7. All enriched GO terms of molecular function for the predicted targets of the selected differentially expressed miRNAs were shown in the Additional file 2. After the cutoff standard of P < 0.05, a total of 21 GO terms of molecular function were found to be associated with cell death and immune regulations (Figure 4). Ten enriched immune- and cell death-related GO terms of molecular function for the miR-147-3p were detected (P < 0.05). These enriched GO terms included programmed cell death, apoptosis, and regulation of T-helper 2 type immune response, lymphocyte activation, lymphocyte differentiation, leukocyte activation, cell death, programmed cell death, apoptosis, and cell proliferation. There were 3, 4, and 4 immune- and cell death-related GO terms of molecular function were enriched for the miR-151-5p, miR-155-3p, and miR-223-3p, respectively. These enriched GO terms included positive regulation of immune system process, response to wounding, regulation of innate immune response, somatic diversification of immune receptors and immunoglobulins, lymphoid progenitor cell differentiation, immunoglobulin V(D)J recombination, reproductive developmental process, regulation of cell death, programmed cell death, and apoptosis (Figure 4).

### Inhibition of miR-147-3p, miR-151-5p, miR-155-3p, and miR-223-3p confers viral pathogenesis in mice

**Figure 5:**
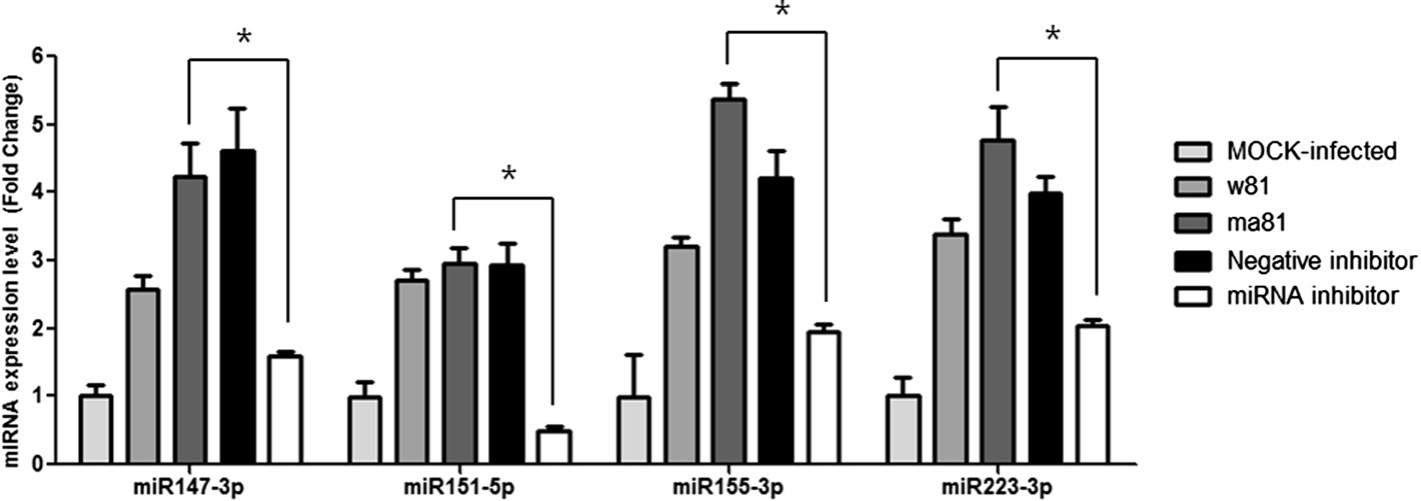
**Inhibition of 4 miRNAs after miRNA inhibitor treatment.** Mock-infected group was inoculated with sterile PBS without any treatment. Mice in w81 and ma81 groups with no treatment were inoculated with w81 and ma81, respectively. Mice in treatment groups were transfected with the individual miRNA inhibitors (Bioneer Co. Ltd, Daejeon, Korea) or negative inhibitor control (miRNA negative inhibitor control #1; Bioneer Co. Ltd, Daejeon, Korea) as indicated, and then intranasally challenged with 5 MLD50 of ma81 H5N2 virus. RNA isolation in mouse lungs (n = 3/group) were conducted at 1 dpi. The miRNA expression levels were measured by qRT-PCR, and were normalized with RNU6. *, P value ≤0.05. Mock-infected: no treatment, then inoculated with sterile PBS. w81: no miRNA inhibitor, then inoculated with w81. ma81: no miRNA inhibitor, then inoculated with ma81. Negative inhibitor: miRNA inhibitor negative control #1 (Bioneer Co. Ltd, Daejeon, Korea) treatment, then inoculated with ma81. miRNA inhibitor: miRNA inhibitor (Bioneer Co. Ltd, Daejeon, Korea) treatment, then inoculated with ma81.

**Figure 6:**
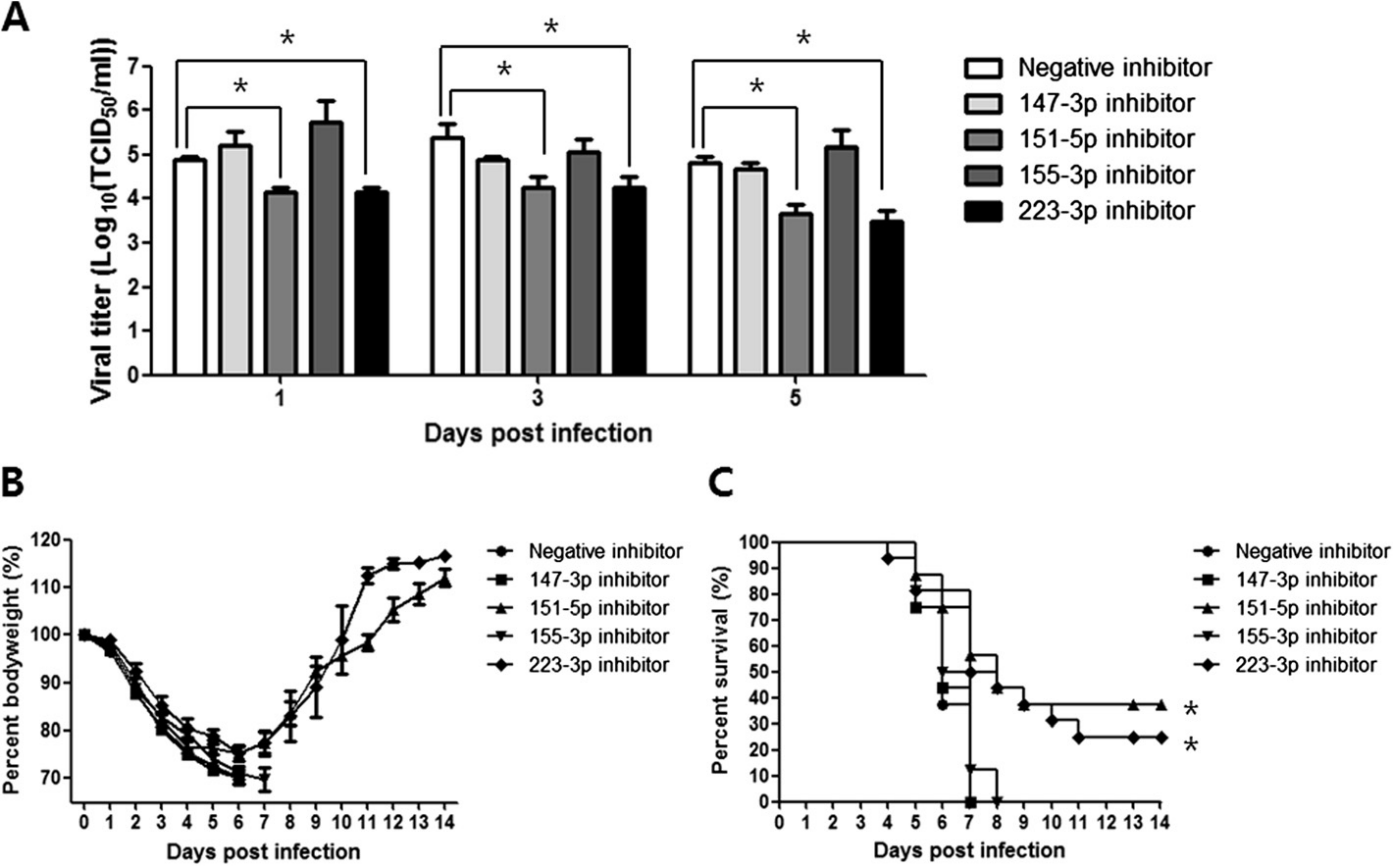
**Impact of miRNA inhibitor transfection on virus infection with invivofectamine.** Groups of 8 (done twice, total 16) or 9 (lung viral titration) C57BL/6 mice were inoculated intranasally with ma81 H5N2 avian influenza virus (5 MLD50) 24 hours after individual transfection of 30 μg miRNA inhibitors (miR-147-3p, miR-151-5p, miR-155-3p, miR-223-3p) or an miRNA negative inhibitor control (miRNA negative inhibitor control #1; Bioneer Co. Ltd, Daejeon, Korea). Viral growth kinetics in mouse lungs were determined by TCID50 at 1, 3, and 5 dpi **(A)**. Data are expressed as mean ± SD titers from 3 lungs per time point. Mean body weight changes **(B)** and survival rates **(C)** were monitored in six remaining mice for 14 days. *, P value ≤0.05.

To investigate the possible roles of miRNAs in virus replication, we generated anti-miRNAs and administered them via intranasal transfection to further evaluate the roles of miR-147-3p, miR-151-5p, miR-155-3p, and miR-223-3p in the pathogenesis of influenza infection in mice. Inhibition of miRNA expressions was examined by real-time RT-PCR. Expression levels of each miRNA in the mouse lungs inoculated with ma81 after transfection of corresponding miRNA inhibitors were significantly decreased compared to those of the lungs inoculated with ma81 without miRNA inhibitor transfection (P < 0.05). In contrast, miRNA negative inhibitor control treatment did not significantly affect the expression level of the miRNAs tested (Figure 5). When viral loads were measured at 1, 3, and 5 dpi, significantly lower influenza virus titers were observed in the lungs of animals treated with anti-miR-151-5p and anti-miR-223-3p than in untreated controls. In addition, the survival rate of the anti-miR-151-5p and anti-miR-223-3p treated mice significantly increased compared to that of the control group (P < 0.05). Approximately 37.5% (6 out of 16) and 25% (4 out of 16) of mice treated with anti-miR-151-5p and anti-miR-223-3p, respectively, survived until the end of the experiment, whereas all untreated control mice were dead by 7 dpi. Moreover, the mice treated with anti-miR-151-5p and anti-miR-223-3p began to gain weight starting at 6 dpi and rapidly recovered their body weight thereafter (Figure 6). Thus, these results indicate that inhibition of miR-151-5p and miR-223-3p reduces influenza replication in the lungs, while the overexpression of these miRNAs in the lungs augments influenza infection. In contrast, all the mice treated with anti-miR-147-3p and anti-miR-155-3p lost body weight as quickly as the control mice and died from influenza infection within 8 dpi. Mice treated with anti-miR-155-3p had a tendency to exhibit higher viral titers than all other groups of mice, although it was not statistically significant compared to negative inhibitor-treated group (P > 0.05) (Figure 6). Therefore, our results indicate that the inhibition of miR-155-3p expression was detrimental for mice during the pathogenesis of influenza infection, suggesting that overexpression of miR-155-3p might exert a protective function against influenza.

## Discussion

MiRNAs have the potential to regulate gene expression through base pairing with complementary sequences in target miRNAs, resulting in translational inhibition or miRNA degradation. Furthermore, it is now acknowledged that the complexity of diverse disease phenotypes, including viral infections, is linked to the altered expression of host miRNAs [42]. Specifically, viruses such as human immunodeficiency virus, respiratory syncytial virus, hepatitis B virus, and Epstein-Barr virus have been shown to alter the expression of host miRNAs [43-46], and miRNA expression profiles have been assessed in the context of various disease phenotypes caused by different influenza strains [47,48]. However, our study is unique in that we looked at the differences in phenotypes through making comparisons between a mouse-adapted variant and its parental virus strain that share relatively small differences in viral genetics. In terms of virulence, the A/Aquatic bird/Korea/ma81/2007 (H5N2) (ma81) variant proved more pathogenic than the parental A/Aquatic bird/Korea/w81/2005 (H5N2) (w81) strain, as all mice infected with the mouse-adapted virus died within 8 dpi while the w81-infected mice survived (Figure 1). This result suggests that even though both viruses have the same genetic backbone, mouse adaptation of an avian influenza virus alters the disease phenotype. In order to determine whether host cell gene expression also differs following infection with these viruses, we compared miRNA expression profiles in mouse lungs using a Solexa deep sequencing approach. In lungs of infected animals, miRNA expression profiles induced by w81 and ma81 were obtained, and differentially expressed miRNAs were compared. While identification of the mechanisms underlying the virulence of ma81 will require further study, the results presented here underscore the feasibility of using miRNAs as prognostic markers or therapeutic targets in the face of a mammalian-adapted, avian influenza virus infection.

A total of 174 and 72 differentially expressed miRNAs were detected in lungs infected with w81 and ma81, respectively (Figure 2). Influenza A virus can alter expressions of numerous miRNAs, and individual miRNAs have the potential to regulate multiple gene expressions. Thus, the complexity of regulation of gene expression caused by miRNAs greatly expands the number of possible virus-host regulatory interactions [47]. Since no global patterns of miRNA expression in response to virus infection have been uncovered, a focus has been placed on the regulation of gene expression by individual miRNAs and their potential roles in disease pathogenesis [46,47,49,50]. Therefore, we also investigated individual miRNAs that were differentially expressed in lungs infected with either w81 or ma81. Among differentially expressed miRNAs, we selected 4 miRNAs with greater than 2-fold differences in expression levels in lungs infected with either w81 or ma81 compared to controls for more in-depth analysis. These miRNAs include miR-151-5p, miR-223-3p, miR-147-3p, and miR-155-3p (Table 5). Potential functions of the selected differentially expressed miRNAs are summarized in Table 5. These 4 miRNAs are known to be involved in cell death or immunity or inflammatory responses. Specifically, these miRNAs have been implicated in immune responses (miR-223-3p and miR-147-3p), viral infection (miR-155-3p), and cell migration (miR-151-5p) [50-56].

In our study, while miR-155-3p and miR-223-3p were highly expressed following infection with both w81 and ma81, their expression was more than 2-fold greater in pathogenic ma81-infected lungs than in w81-infected lungs. Previous studies on expression of miR-155 and miR-223-3p in relation to immune responses and viral infections [52,55-58] have reported that miR-155-3p expression may inhibit malignant growth and viral infections [51,52,54,55]. A role for miR-155 in the mammalian immune system was suggested by the finding that immune responses are defective in miR-155 knockout mice due to altered lymphocyte differentiation, which is likely a result of disregulated cytokine production [58]. In accordance with these findings, our results demonstrate that the inhibition of miR-155-3p expression was detrimental to mice during the pathogenesis of influenza infection. Specifically, mice treated with anti-miR-155-3p rapidly lost body weight and died from influenza infection within 8 dpi, and they had higher viral titers in the lungs than other groups of mice (Figure 6). Although the wide range of biological functions attributed to each miRNA during influenza infection precludes the conclusion that miR-155-3p is responsible for induction of the cellular antiviral effect, the results from our study along with those from Thai et al., at least, indicate that overexpression of miR-155-3p is one of the defense mechanisms working against influenza infection [58]. Another study suggested the possible regulation of influenza virus infection by miR-155 in chickens due to its targeting of the chicken anti-influenza gene MX1 and activation of the JUK pathway [28].

Multiple functions for miR-223-3p have been previously reported [56], and miR-223-3p is known to promote granulocytic differentiation [53,56]. The current study confirms highly upregulated expression of miR-223-3p during influenza A virus infections, which has previously been reported following infection with pathogenic 1918 pandemic H1N1 influenza in mouse lungs infected with H5N1 and in pig lungs infected with H1N2 [23,26,27,48,59]. Additionally, significantly lower influenza virus titers were measured in the influenza-infected lungs treated with anti-miR-223-3p than in non-treated lungs. In addition, 25% of mice treated with anti-miR-223-3p regained their body weight and survived lethal influenza infection (Figure 6). These results indicate that overexpression of miR-223-3p is detrimental to the host during influenza infection. Thus, inhibition of miR-223-3p might reduce the pathogenicity of this influenza virus in the host during infection. Although identification of the precise roles of miR-155-3p and miR-223-3p during influenza infection will require further study, based on the results of this study and several others, it is apparent that miR-155-3p and miR-223-3p contribute to pathogenicity [23,26,27,48,58].

Potential roles of miR-151-5p may be related to the previously identified function of this molecule in the regulation of tumor cell migration and spreading of hepatocellular carcinoma [60]. Ding et al. highlighted the detrimental role of overexpressed miR-151-5p in the pathogenesis of hepatocellular carcinoma (HCC). In their study, significantly increased HCC cell migration and invasion were observed when miR-151-5p, together with its host gene (encoding focal adhesion kinase), was overexpressed. In our study, the expression of miR-151-5p in lungs infected with the influenza virus was found to be upregulated at all time points examined. Furthermore, inhibition of miR-151-5p expression reduced influenza virus titers compared to those of infected lungs from mice not treated with the inhibitor, and 30% of mice treated with anti-miR-151-5p regained their body weight and survived influenza infection (Figure 6). Altogether, we can speculate that the silencing of miR-151-5p expression alters the pathogenesis of the influenza virus and promotes the antiviral ability of the host during influenza infection. In this study, the expression of miR-151-5p was found to be upregulated at all time points assessed, in contrast to reports that its expression was downregulated in response to infection with H1N1 and H5N1 [25,61]. This discrepancy may be due to the different experimental design. For example, we used lung tissues to evaluate expression of each miRNA, while Tambyah et al. analyzed miRNA expression levels in human blood. Also, avian influenza virus was inoculated into Myxovirus resistance (Mx1) protein-deficient C57BL/6 mice in this study because avian strains are more susceptible to the antiviral effect of Mx proteins, while the other studies used human influenza virus and different animals.

In addition, expression of miR-147-3p was upregulated at all times in lungs infected with either w81 or ma81. MiR-147-3p has been shown to be associated with Toll-like receptor stimulation and regulation of murine macrophage inflammatory responses [62,63]. Even with their known immunological functions (Table 5), downregulation of miR-147-3p or miR-155-3p by each miRNA inhibitor could not alter the viral replication and mortality in infected mice. Therefore, elucidation of the potential roles of miR-147-3p in host cells during influenza infection will require further study.

## Conclusions

The importance of miRNAs as gene regulators in the host during influenza infections has been broadly defined, although the exact functions of these molecules in viral pathogenesis remain to be determined. The study presented here highlights differentially expressed miRNAs in mouse lungs infected with wild type, low pathogenic H5N2, and its mouse-adapted, highly pathogenic variant. Furthermore, this study suggests that some of the miRNAs identified are capable of boosting the pathogenesis of influenza. Thus, some of these miRNAs may be useful as potential prognostic or therapeutic targets in the face of avian influenza A virus infections in mammalian hosts.

## Methods

### Virus and cell

Virus strain A/Aquatic bird/Korea/w81/05 (H5N2) (w81) was isolated from wild birds. A/Aquatic bird/Korea/ma81/07 virus was derived from w81 (H5N2) (ma81), mouse-adapted highly pathogenic H5N2 [41]. Both w81 and ma81 were propagated and maintained in Madin-Darby canine kidney (MDCK) cells in Eagle’s Minimum Essential Medium (EMEM), supplemented with 5% fetal bovine serum (FBS) and incubated at 37°C in 5% CO2.

### Mouse experiments and viral titration in lungs for pathogenicity of ma81

C57BL/6 female mice (5-week-old) were anesthetized with ketamine-xylazine and infected by intranasal instillation with 30 ul of 10^4^ TCID_50_ of w81 or ma81. Lung tissue from 3 mice per group was harvested on 1, 3, 5, and 7 days post infection (dpi). To assess survival, groups of 6 mice were inoculated with virus and monitored daily for 14 days. Viral load in mouse lungs was titrated on MDCK cells as previously described [64].

### RNA isolation from lung tissue and miRNA Deep Sequencing

For total RNA extraction, entire lungs from mice infected by w81 or ma81 were harvested at 1 and 3 dpi, 3 lungs per time point. Total RNAs of lung samples were isolated using Trizol (Invitrogen, Carlsbad, CA), RNeasy Total RNA Kit (Qiagen, Chatsworth, CA, USA) according to manufacturer’s protocol.

A small RNA library was constructed and sequenced at Theragen (Suwon, Korea) using the Solexa high-throughput platform [Illumina, San Diego, CA]. In brief, this consisted of gel purification of the RNA bands corresponding to size fractionation of 18-30 nt, ligation of adapters to 3’ and 5’ regions of small RNA using T4-RNA ligase, cDNA synthesis using RT-PCR, and final PCR amplification for Solexa sequencing.

### MiRNA analysis

To minimize the effects of random sequencing errors, we eliminated sequences with <18 bases and sequences that contained more than 1 undetermined nucleotide (N). Sequencing quality (sQ) was evaluated as follows, where E indicates sequencing error rate [Illumina, San Diego, CA]:

$$ \mathrm{s}\mathrm{Q}=-10\times \raisebox{1ex}{$ log\frac{E}{1-E}$}\!\left/ \!\raisebox{-1ex}{$ log10$}\right. $$

Sequences having more than 6 bases with *sQ* value of < 13 were discarded. The resulting quality-controlled sequence reads were mapped to the database, miRNA precursor/mature of mouse, in miRBase 15.0 and Genebank using the SOAP alignment program [65]. Complete alignment of the sequences was required and no mismatches were allowed. We compared the known miRNA expression levels between 2 treatment samples to identify the differentially expressed miRNAs. Briefly, sequence reads were normalized to determine the number of transcripts per million (TPM) using the following formula: Normalized expression = Actual miRNA count/Total count of clean reads*1000000. Then, fold changes of miRNAs were evaluated using the following formula: Fold change = log2(treatment/control). P-value was calculated from the normalized expression values using the following formula:

P-value formula [66]: x, y, N1 and N2 represent number of miRNAs surveyed, number of homologous miRNAs in controls, total number of clean reads in controls, and total number of clean reads in treatments, respectively.

$$ \mathrm{p}\left(\mathrm{x}\Big|\mathrm{y}\right)=\left(\frac{{\mathrm{N}}_2}{{\mathrm{N}}_1}\right)\frac{\left(\mathrm{x}+\mathrm{y}\right)!}{\mathrm{x}!\mathrm{y}!{\left(1+\frac{{\mathrm{N}}_2}{{\mathrm{N}}_1}\right)}^{\left(\mathrm{x}+\mathrm{y}+1\right)}} $$

$$ \mathrm{c}\left(\left.\mathrm{y}\le {\mathrm{y}}_{\min}\right|\mathrm{x}\right)={\displaystyle \sum_{\mathrm{y}=0}^{\mathrm{y}\le {\mathrm{y}}_{\min }}p\left(\left.\mathrm{y}\right|\mathrm{x}\right)} $$

$$ \mathrm{c}\left(\left.\mathrm{y}\le {\mathrm{y}}_{\max}\right|\mathrm{x}\right)={\displaystyle \sum_{\mathrm{y}\le {\mathrm{y}}_{\min}}^{\infty}\mathrm{p}\left(\left.\mathrm{y}\right|\mathrm{x}\right)} $$

### Confirmation of miRNA expression profiles by quantitative real-time PCR

Quantitative real-time PCR was used to validate miRNA expression using the same total RNA samples for small RNA library constructions. Briefly, cDNA was synthesized by using an miScriptII RT Kit (Qiagen, Hilden, Germany). qRT-PCR was performed using miScript SYBR Green PCR Kit (Qiagen, Hilden, Germany) on a Rotor Gene RG-3000 (Corbett Research, Sydney, Australia). The following primer sets were purchased from the miScript Primer Assays (Qiagen, Hilden, Germany) and used in this study: mmu-miR-147-3p, mmu-miR-151-5p, mmu-miR-155-3p, and mmu-miR-223-3p. Cycling conditions were 95°C for 15 min followed by 45 cycles at 94°C for 15 sec, 55°C for 30 sec, and 70°C for 30 sec. U6 was used for normalization. Data were analyzed using the 2-△△Ct PCR.

### Gene ontology analysis

Gene ontology analysis was conducted as previously described [28]. Briefly, miRanda version 3.0 was used to predict potential target genes of 4 miRNAs with greater than 2-fold differences between expression levels in lungs infected with either w81 or ma81 compared to the control [67]. Then all target genes of each miRNA were used for the gene ontology (GO) analysis using DAVID version 6.7 [68]. Functional category enrichment was evaluated based on the GO terms of each miRNA. The enrichment of GO terms was selected with a cutoff standard of P < 0.05.

### MiRNA inhibition

Based on the original miRNA sequences, all inhibitors were designed and synthesized by Bioneer Co. Ltd (Daejeon, Korea). Groups of mice (n = 25) were transfected with 30 ug of miRNA inhibitors (miR-147-3p, miR-151-5p, miR-155-3p, miR-223-3p) or an miRNA negative inhibitor control (miRNA negative inhibitor control #1; Bioneer Co. Ltd, Daejeon, Korea). Briefly, each miRNA inhibitor (3 mg/ml) was mixed with invivofectamine complexation buffer and reagent (Invitrogen, Life technologies Corporation, USA), then the mixed solution was transferred to a pre-washed Amicon Ultra-15 centrifugal tube, which was centrifuged at 4000xg for 30 min according to manufacturer protocols. The final concentration of each inhibitor was 1.5 ug/ul, and mice were transfected intranasally with 30 ug of each inhibitor.

On day after infection, miRNA inhibitor treated mice were infected by intranasal instillation with 30 ul of 104 TCID50 of ma81 virus. Lung tissues from 3 mice per group were harvested on 1, 3, and 5 days post infection (dpi) for viral titration, and survival rates were monitored daily for 14 days.

### Statistical analysis

The Mantel-Cox log rank test implemented in GraphPad Prism software version 5 (GraphPad Software, La Jolla, CA) was used for the survival analysis, and Student’s t-test were used to compare viral titers, percent bodyweight, and expression levels of miRNAs between groups.

### Ethics statement

The study protocols for the use of mice were carried out in strict accordance and adherence to relevant policies regarding animal handling as mandated under the Guidelines for Animal Use and Care of the Korea Center for Disease Control (K-CDC) and were approved by the Medical Research Institute of Chungbuk National University (approval number CBNU-IRB-2012-GM01).

## Additional files

Additional file 1:
**Expression level of 4 miRNAs was verified by qRT-PCR.** Groups of 3 (done twice, total 6) C57BL/6 mice were inoculated intranasally with 5 MLD50 of w81 or ma81 H5N2 avian influenza virus. RNA isolation in mouse lungs were conducted at 1 and 3dpi, and then the miRNA expression levels were measured by qRT-PCR in triplicate. The similar results confirmed the upregulation of 4 miRNAs with deep sequencing results. *, P value ≤0.05. Additional file 2:
**GO terms of molecular function for the predicted targets of the selected miRNAs.** Potential target genes of 4 selected miRNAs were predicted using miRanda version 3.0. Then all target genes of each miRNA were used for the gene ontology (GO) analysis using DAVID version 6.7.

## Abbreviations

AI: Avian influenza
miRNA: MicroRNA
ma: Mouse adapted
GO: Gene ontology
MLD50: Mouse lethal dose 50%
dpi: Days post infection
Mx1: Myxovirus resistance
TCID50: 50% Tissue culture infectious doses
MDCK: Madin-Darby canine kidney
EMEM: Eagle’s minimum essential medium
FBS: Fetal bovine serum
N: Nucleotide
Sq: Sequencing quality
TPM: Transcripts per million

## References

[1] Lamb RA, Krug RM, Knipe DM, Howley PM, Lamb RA, Martin MA, Roizman B, Straus SE (2001). Orthomyxoviridae: The viruses and their replication. Fields Virology. Volume 1.

[2] Kawaoka Y, Chambers TM, Sladen WL, Webster RG (1988). Is the gene pool of influenza viruses in shorebirds and gulls different from that in wild ducks?. Virology.

[3] Hinshaw VS, Webster RG, Beare AS (1982). The natural history of influenza A viruses. Basic and Applied Influenza Research.

[4] Gao S, Song L, Li J, Zhang Z, Peng H, Jiang W, Wang Q, Kang T, Chen S, Huang W (2012). Influenza A virus-encoded NS1 virulence factor protein inhibits innate immune response by targeting IKK. Cell Microbiol.

[5] Wise HM, Foeglein A, Sun J, Dalton RM, Patel S, Howard W, Anderson EC, Barclay WS, Digard P (2009). A complicated message: Identification of a novel PB1-related protein translated from influenza A virus segment 2 mRNA. J Virol.

[6] Palese P, Shaw ML, Knipe DM, Howley PM, Griffin DE (2007). Orthomyxoviridae: The viruses and their Replication. Fields Virology. Volume 2.

[7] Webster RG, Bean WJ, Gorman OT, Chambers TM, Kawaoka Y (1992). Evolution and ecology of influenza A viruses. Microbiol Rev.

[8] Johnson NP, Mueller J (2002). Updating the accounts: global mortality of the 1918–1920 “Spanish” influenza pandemic. Bull Hist Med.

[9] Perrone LA, Plowden JK, Garcia-Sastre A, Katz JM, Tumpey TM (2008). H5N1 and 1918 pandemic influenza virus infection results in early and excessive infiltration of macrophages and neutrophils in the lungs of mice. PLoS Pathog.

[10] Itoh Y, Shinya K, Kiso M, Watanabe T, Sakoda Y, Hatta M, Muramoto Y, Tamura D, Sakai-Tagawa Y, Noda T, Sakabe S, Imai M, Hatta Y, Watanabe S, Li C, Yamada S, Fujii K, Murakami S, Imai H, Kakugawa S, Ito M, Takano R, Iwatsuki-Horimoto K, Shimojima M, Horimoto T, Goto H, Takahashi K, Makino A, Ishigaki H, Nakayama M (2009). In vitro and in vivo characterization of new swine-origin H1N1 influenza viruses. Nature.

[11] Kobasa D, Jones SM, Shinya K, Kash JC, Copps J, Ebihara H, Hatta Y, Kim JH, Halfmann P, Hatta M, Feldmann F, Alimonti JB, Fernando L, Li Y, Katze MG, Feldmann H, Kawaoka Y (2007). Aberrant innate immune response in lethal infection of macaques with the 1918 influenza virus. Nature.

[12] de Jong MD, Simmons CP, Thanh TT, Hien VM, Smith GJ, Chau TN, Hoang DM, Chau NV, Khanh TH, Dong VC, Qui PT, Cam BV, Ha DQ, Guan Y, Peiris JS, Chinh NT, Hien TT, Farrar J (2006). Fatal outcome of human influenza A (H5N1) is associated with high viral load and hypercytokinemia. Nat Med.

[13] Szretter KJ, Gangappa S, Lu X, Smith C, Shieh WJ, Zaki SR, Sambhara S, Tumpey TM, Katz JM (2007). Role of host cytokine responses in the pathogenesis of avian H5N1 influenza viruses in mice. J Virol.

[14] Bartel DP (2004). MicroRNAs: genomics, biogenesis, mechanism, and function. Cell.

[15] Grassmann R, Jeang KT (2008). The roles of microRNAs in mammalian virus infection. Biochim Biophys Acta.

[16] Ghosh Z, Mallick B, Chakrabarti J (2009). Cellular versus viral microRNAs in host-virus interaction. Nucleic Acids Res.

[17] Gaulke CA, Porter M, Han YH, Sankaran-Walters S, Grishina I, George MD, Dang AT, Ding SW, Jiang G, Korf I, Dandekar S (2014). Intestinal epithelial barrier disruption through altered mucosal microRNA expression in human immunodeficiency virus and simian immunodeficiency virus infections. J Virol.

[18] Houzet L, Yeung ML, de Lame V, Desai D, Smith SM, Jeang KT (2008). MicroRNA profile changes in human immunodeficiency virus type 1 (HIV-1) seropositive individuals. Retrovirology.

[19] Pan XB, Ma H, Jin Q, Wei L (2012). Characterization of microRNA expression profiles associated with hepatitis B virus replication and clearance in vivo and in vitro. J Gastroenterol Hepatol.

[20] Liu X, Wang T, Wakita T, Yang W (2010). Systematic identification of microRNA and messenger RNA profiles in hepatitis C virus-infected human hepatoma cells. Virology.

[21] Imig J, Motsch N, Zhu JY, Barth S, Okoniewski M, Reineke T, Tinguely M, Faggioni A, Trivedi P, Meister G, Renner C, Grässer FA (2011). microRNA profiling in Epstein-Barr virus-associated B-cell lymphoma. Nucleic Acids Res.

[22] Wang X, Wang HK, Li Y, Hafner M, Banerjee NS, Tang S, Briskin D, Meyers C, Chow LT, Xie X, Tuschl T, Zhenga ZM (2014). microRNAs are biomarkers of oncogenic human papillomavirus infections. Proc Natl Acad Sci U S A.

[23] Li Y, Chan EY, Li J, Ni C, Peng X, Rosenzweig E, Tumpey TM, Katze MG (2010). MicroRNA expression and virulence in pandemic influenza virus-infected mice. J Virol.

[24] Loveday EK, Svinti V, Diederich S, Pasick J, Jean F (2012). Temporal- and strain-specific host microRNA molecular signatures associated with swine-origin H1N1 and avian-origin H7N7 influenza A virus infection. J Virol.

[25] Li Y, Li J, Belisle S, Baskin CR, Tumpey TM, Katze MG (2011). Differential microRNA expression and virulence of avian, 1918 reassortant, and reconstructed 1918 influenza A viruses. Virology.

[26] Rogers JV, Price JA, Wendling MQ, Long JP, Bresler HS (2012). Preliminary microRNA analysis in lung tissue to identify potential therapeutic targets against H5N1 infection. Viral Immunol.

[27] Skovgaard K, Cirera S, Vasby D, Podolska A, Breum SO, Durrwald R, Schlegel M, Heegaard PM (2013). Expression of innate immune genes, proteins and microRNAs in lung tissue of pigs infected experimentally with influenza virus (H1N2). Innate Immun.

[28] Wang Y, Brahmakshatriya V, Zhu H, Lupiani B, Reddy SM, Yoon BJ, Gunaratne PH, Kim JH, Chen R, Wang J, Zhou H (2009). Identification of differentially expressed miRNAs in chicken lung and trachea with avian influenza virus infection by a deep sequencing approach. BMC Genomics.

[29] Wang Y, Brahmakshatriya V, Lupiani B, Reddy SM, Soibam B, Benham AL, Gunaratne P, Liu HC, Trakooljul N, Ing N, Okimoto R, Zhou H (2012). Integrated analysis of microRNA expression and mRNA transcriptome in lungs of avian influenza virus infected broilers. BMC Genomics.

[30] Terrier O, Textoris J, Carron C, Marcel V, Bourdon JC, Rosa-Calatrava M (2013). Host microRNA molecular signatures associated with human H1N1 and H3N2 influenza A viruses reveal an unanticipated antiviral activity for miR-146a. J Gen Virol.

[31] Song L, Liu H, Gao S, Jiang W, Huang W (2010). Cellular microRNAs inhibit replication of the H1N1 influenza A virus in infected cells. J Virol.

[32] Brown EG, Bailly JE (1999). Genetic analysis of mouse-adapted influenza A virus identifies roles for the NA, PB1, and PB2 genes in virulence. Virus Res.

[33] Brown EG, Liu H, Kit LC, Baird S, Nesrallah M (2001). Pattern of mutation in the genome of influenza A virus on adaptation to increased virulence in the mouse lung: identification of functional themes. Proc Natl Acad Sci U S A.

[34] Kaverin NV, Finskaya NN, Rudneva IA, Gitelman AK, Kharitonenkov IG, Smirnov YA (1989). Studies on the genetic basis of human influenza A virus adaptation to mice: degrees of virulence of reassortants with defined genetic content. Arch Virol.

[35] Rudneva IA, Kaverin NV, Varich NL, Gitelman AK, Makhov AM, Klimenko SM, Zhdanov VM (1986). Studies on the genetic determinants of influenza virus pathogenicity for mice with the use of reassortants between mouse-adapted and non-adapted variants of the same virus strain. Arch Virol.

[36] Smeenk CA, Brown EG (1994). The influenza virus variant A/FM/1/47-MA possesses single amino acid replacements in the hemagglutinin, controlling virulence, and in the matrix protein, controlling virulence as well as growth. J Virol.

[37] Ward AC (1996). Neurovirulence of influenza A virus. J Neurovirol.

[38] Garten RJ, Davis CT, Russell CA, Shu B, Lindstrom S, Balish A, Sessions WM, Xu X, Skepner E, Deyde V, Okomo-Adhiambo M, Gubareva L, Barnes J, Smith CB, Emery SL, Hillman MJ, Rivailler P, Smagala J, de Graaf M, Burke DF, Fouchier RA, Pappas C, Alpuche-Aranda CM, López-Gatell H, Olivera H, López I, Myers CA, Faix D, Blair PJ, Yu C (2009). Antigenic and genetic characteristics of swine-origin 2009 A(H1N1) influenza viruses circulating in humans. Science.

[39] Hatta M, Gao P, Halfmann P, Kawaoka Y (2001). Molecular basis for high virulence of Hong Kong H5N1 influenza A viruses. Science.

[40] Munster VJ, de Wit E, van Riel D, Beyer WE, Rimmelzwaan GF, Osterhaus AD, Kuiken T, Fouchier RA (2007). The molecular basis of the pathogenicity of the Dutch highly pathogenic human influenza A H7N7 viruses. J Infect Dis.

[41] Song MS, Pascua PN, Lee JH, Baek YH, Lee OJ, Kim CJ, Kim H, Webby RJ, Webster RG, Choi YK (2009). The polymerase acidic protein gene of influenza a virus contributes to pathogenicity in a mouse model. J Virol.

[42] Gottwein E (2013). Roles of microRNAs in the life cycles of mammalian viruses. Curr Top Microbiol Immunol.

[43] Onnis A, Navari M, Antonicelli G, Morettini F, Mannucci S, De FG, Vigorito E, Leoncini L (2012). Epstein-Barr nuclear antigen 1 induces expression of the cellular microRNA hsa-miR-127 and impairing B-cell differentiation in EBV-infected memory B cells. New insights into the pathogenesis of Burkitt lymphoma. Blood Cancer J.

[44] Gupta P, Liu B, Wu JQ, Soriano V, Vispo E, Carroll AP, Goldie BJ, Cairns MJ, Saksena NK (2014). Genome-wide mRNA and miRNA analysis of peripheral blood mononuclear cells (PBMC) reveals different miRNAs regulating HIV/HCV co-infection. Virology.

[45] Bakre A, Mitchell P, Coleman JK, Jones LP, Saavedra G, Teng M, Tompkins SM, Tripp RA (2012). Respiratory syncytial virus modifies microRNAs regulating host genes that affect virus replication. J Gen Virol.

[46] Zou C, Li Y, Cao Y, Zhang J, Jiang J, Sheng Y, Wang S, Huang A, Tang H (2014). Up-regulated MicroRNA-181a induces carcinogenesis in Hepatitis B virus-related hepatocellular carcinoma by targeting E2F5. BMC Cancer.

[47] Lam WY, Yeung AC, Ngai KL, Li MS, To KF, Tsui SK, Chan PK (2013). Effect of avian influenza A H5N1 infection on the expression of microRNA-141 in human respiratory epithelial cells. BMC Microbiol.

[48] Wu Z, Hao R, Li P, Zhang X, Liu N, Qiu S, Wang L, Wang Y, Xue W, Liu K, Yang G, Cui J, Zhang C, Song H (2013). MicroRNA expression profile of mouse lung infected with 2009 pandemic H1N1 influenza virus. PLoS One.

[49] Liu G, Friggeri A, Yang Y, Park YJ, Tsuruta Y, Abraham E (2009). miR-147, a microRNA that is induced upon Toll-like receptor stimulation, regulates murine macrophage inflammatory responses. Proc Natl Acad Sci U S A.

[50] Luedde T (2010). MicroRNA-151 and its hosting gene FAK (focal adhesion kinase) regulate tumor cell migration and spreading of hepatocellular carcinoma. Hepatology.

[51] Mattiske S, Suetani RJ, Neilsen PM, Callen DF (2012). The oncogenic role of miR-155 in breast cancer. Cancer Epidemiol Biomarkers Prev.

[52] Vargova K, Curik N, Burda P, Basova P, Kulvait V, Pospisil V, Savvulidi F, Kokavec J, Necas E, Berkova A, Obrtlikova P, Karban J, Mraz M, Pospisilova S, Mayer J, Trneny M, Zavadil J, Stopka T (2011). MYB transcriptionally regulates the miR-155 host gene in chronic lymphocytic leukemia. Blood.

[53] Johnnidis JB, Harris MH, Wheeler RT, Stehling-Sun S, Lam MH, Kirak O, Brummelkamp TR, Fleming MD, Camargo FD (2008). Regulation of progenitor cell proliferation and granulocyte function by microRNA-223. Nature.

[54] Wang L, Toomey NL, Diaz LA, Walker G, Ramos JC, Barber GN, Ning S (2011). Oncogenic IRFs provide a survival advantage for Epstein-Barr virus- or human T-cell leukemia virus type 1-transformed cells through induction of BIC expression. J Virol.

[55] Babar IA, Cheng CJ, Booth CJ, Liang X, Weidhaas JB, Saltzman WM, Slack FJ (2012). Nanoparticle-based therapy in an in vivo microRNA-155 (miR-155)-dependent mouse model of lymphoma. Proc Natl Acad Sci U S A.

[56] Fazi F, Racanicchi S, Zardo G, Starnes LM, Mancini M, Travaglini L, Diverio D, Ammatuna E, Cimino G, Lo-Coco F, Grignani F, Nervi C (2007). Epigenetic silencing of the myelopoiesis regulator microRNA-223 by the AML1/ETO oncoprotein. Cancer Cell.

[57] Sonkoly E, Stahle M, Pivarcsi A (2008). MicroRNAs and immunity: novel players in the regulation of normal immune function and inflammation. Semin Cancer Biol.

[58] Thai TH, Calado DP, Casola S, Ansel KM, Xiao C, Xue Y, Murphy A, Frendewey D, Valenzuela D, Kutok JL, Schmidt-Supprian M, Rajewsky N, Yancopoulos G, Rao A, Rajewsky K (2007). Regulation of the germinal center response by microRNA-155. Science.

[59] Rangrez AY, M’Baya-Moutoula E, Metzinger-Le MV, Henaut L, Djelouat MS, Benchitrit J, Massy ZA, Metzinger L (2012). Inorganic phosphate accelerates the migration of vascular smooth muscle cells: evidence for the involvement of miR-223. PLoS One.

[60] Ding J, Huang S, Wu S, Zhao Y, Liang L, Yan M, Ge C, Yao J, Chen T, Wan D, Wang H, Gu J, Yao M, Li J, Tu H, He X (2010). Gain of miR-151 on chromosome 8q24.3 facilitates tumour cell migration and spreading through downregulating RhoGDIA. Nat Cell Biol.

[61] Tambyah PA, Sepramaniam S, Mohamed AJ, Chai SC, Swaminathan P, Armugam A, Jeyaseelan K (2013). microRNAs in circulation are altered in response to influenza A virus infection in humans. PLoS One.

[62] Taganov KD, Boldin MP, Chang KJ, Baltimore D (2006). NF-kappaB-dependent induction of microRNA miR-146, an inhibitor targeted to signaling proteins of innate immune responses. Proc Natl Acad Sci U S A.

[63] Hummel R, Hussey DJ, Haier J (2010). MicroRNAs: predictors and modifiers of chemo- and radiotherapy in different tumour types. Eur J Cancer.

[64] Park SJ, Lee EH, Choi EH, Pascua PN, Kwon HI, Kim EH, Lim GJ, Decano A, Kim SM, Choi YK (2014). Avian-derived NS gene segments alter pathogenicity of the A/Puerto Rico/8/34 virus. Virus Res.

[65] Li R, Li Y, Kristiansen K, Wang J (2008). SOAP: short oligonucleotide alignment program. Bioinformatics.

[66] Peng T, Lv Q, Zhang J, Li J, Du Y, Zhao Q (2011). Differential expression of the microRNAs in superior and inferior spikelets in rice (Oryza sativa). J Exp Bot.

[67] John B, Enright AJ, Aravin A, Tuschl T, Sander C, Marks DS (2004). Human MicroRNA targets. PLoS Biol.

[68] Dennis G, Sherman BT, Hosack DA, Yang J, Gao W, Lane HC, Lempicki RA (2003). DAVID: Database for Annotation, Visualization, and Integrated Discovery. Genome Biol.

