# Supplementary figures and images for: Differential microRNA expression following infection with a mouse-adapted, highly virulent avian H5N2 virus
Source: BMC Microbiol. 2014 Sep 30;14:252. doi: 10.1186/s12866-014-0252-0 (PMC4189662; doi:10.1186/s12866-014-0252-0)

## Slide 1
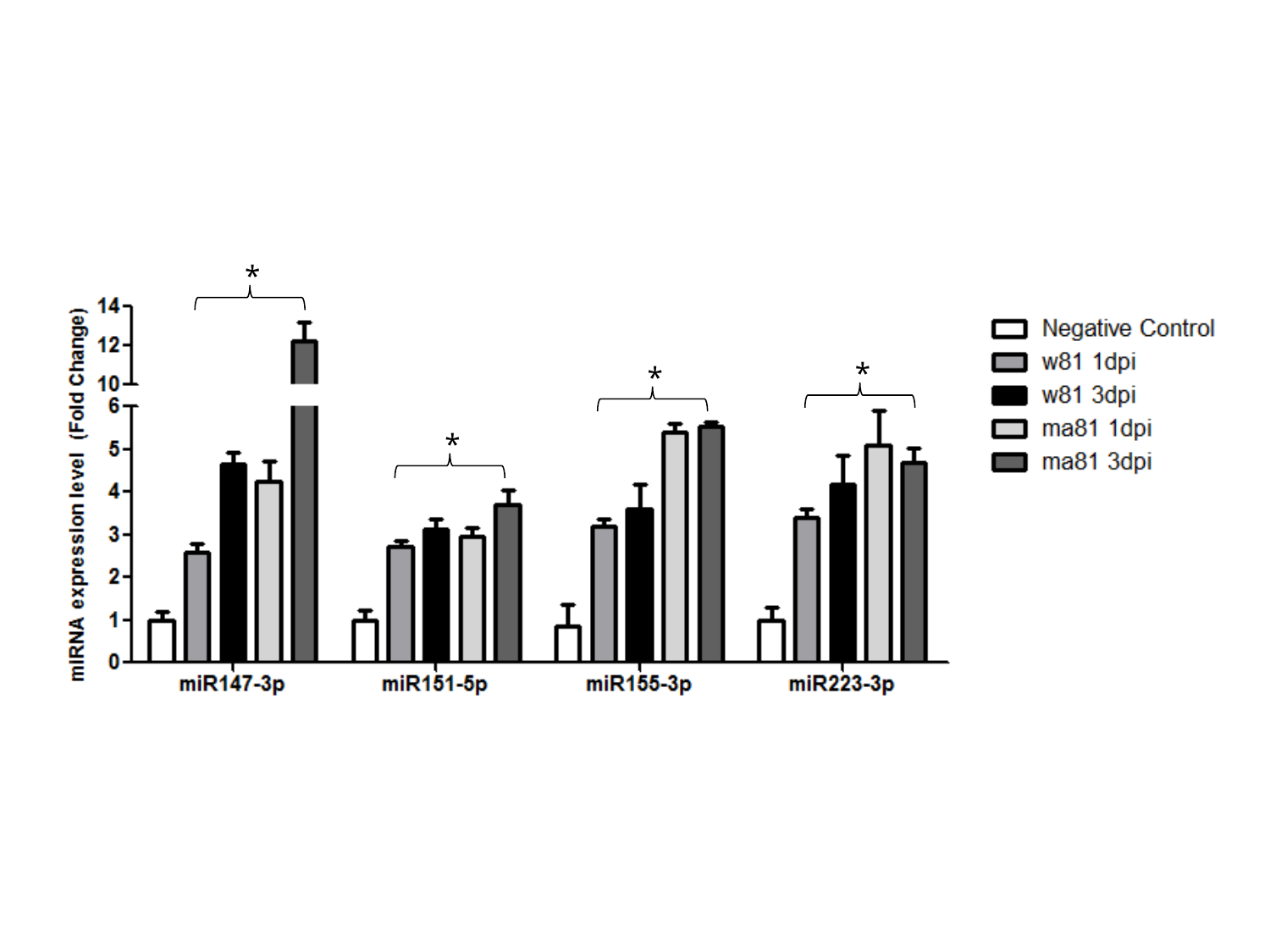

*
*
*
*

Supplement: Additional file 1: — Expression level of 4 miRNAs was verified by qRT-PCR. Groups of 3 (done twice, total 6) C57BL/6 mice were inoculated intranasally with 5 MLD50 of w81 or ma81 H5N2 avian influenza virus. RNA isolation in mouse lungs were conducted at 1 and 3dpi, and then the miRNA expression levels were measured by qRT-PCR in triplicate. The similar results confirmed the upregulation of 4 miRNAs with deep sequencing results. *, P value ≤0.05. [file 12866_2014_252_MOESM1_ESM.ppt]
